# Beneficial effect of Arbidol in the management of COVID-19 infection

**DOI:** 10.18632/aging.202867

**Published:** 2021-04-03

**Authors:** Xiong Jie, Yuan Hongmei, Fan Ping, Zhu Kuikui, Yang Bohan, Meng Rui

**Affiliations:** 1 Cancer Center, Union Hospital, Tongji Medical College, Huazhong University of Science and Technology, Wuhan 430022, China; 2 Department of Pathology, Wuhan Jinyintan Hospital, Wuhan 430023, Hubei, China; 3 Department of Respiratory and Critical Care Medicine, Union Hospital, Tongji Medical College, Huazhong University of Science and Technology, Wuhan 430022, China

**Keywords:** COVID-19, pneumonia, Arbidol, therapeutics

## Abstract

This study analyzed the effect of Arbidol, a broad-spectrum antiviral compound, on the outcomes of COVID-19 patients. Records of 252 COVID-19 patients were retrospectively analyzed from February 13 to February 29, 2020 in 4 inpatient wards in the Cancer Center, Union Hospital, Tongji Medical College of Huazhong University of Science and Technology, Wuhan, China. The rate of clinical improvement was significantly greater among patients treated with Arbidol than among those who did not receive Arbidol (86.8% vs. 54.2%). In moderately and severely ill patients, the clinical improvement rates in the Arbidol group were 95.6% and 81.7%, respectively, which was significantly higher than in the no-Arbidol group (66.6% and 53.8%). Among critically ill patients, however, there was no significant difference. The levels of hypersensitive C-reactive protein, lactate dehydrogenase, D-dimer, IL-6, and IL-10 were increased in non-improved patients but declined during treatment in the improved patients. This suggests these mediators are associated with the disease severity and could potentially serve as prognostic markers. Moreover, our data demonstrate that Arbidol is effective in the treatment of COVID-19 patients and may serve as a cost-effective antiviral treatment strategy for patients with moderate to severe COVID-19 symptoms.

## INTRODUCTION

In December 2019, a group of patients in Wuhan, China, who were related to the Huanan seafood market, presented with pneumonia of an unknown cause. Gene sequencing of the virus isolated from these patients revealed infection with a novel type of coronavirus, named as 2019 novel coronavirus (2019-nCoV), or severe acute respiratory syndrome coronavirus 2 (SARS-CoV-2). Coronavirus disease 2019 (COVID-19) soon drew a global attention and caused a pandemic in the world [1–4]. According to the World Health Organization, there have been 73, 275, 943 confirmed cases of COVID-19 in the world, including 1,650,348 deaths, by December 18, 2020.

The current management of COVID-19 is mainly supportive care, and some anti-virus drugs maybe effective for SARS-CoV-2 [5, 6]. However, there is no consensus on the selection of antiviral drugs, and clinical treatment experience is limited. Arbidol is a small indole-derivative molecule, licensed in Russia and China for prophylaxis and treatment of influenza and other respiratory viral infections [7]. Arbidol demonstrates inhibitory activity against several enveloped and non-enveloped RNA and DNA viruses, including hepatitis B and C, respiratory syncytial virus, severe acute respiratory syndrome coronavirus (SARS-CoV), and middle east respiratory syndrome coronavirus (MERS-CoV) [7, 8]. SARS-CoV-2 is about 78% homologous to SARS-CoV and 58% to MERS-CoV [9]. Previous studies showed that Arbidol could inhibit SARS-CoV-2 *in vitro*; at 10–30 micromolar concentration, Arbidol inhibited the virus up to 60 times, and suppressed the viral pathological effect on cells [10]. Thus, Arbidol was listed as an early anti-viral treatment option in “The fifth edition of the China Guidelines for the Diagnosis and Treatment Plan of Novel Coronavirus (2019-nCoV) Infection (Trial Version 5)” issued by the National Health Commission of China [11]. However, evidence for beneficial effects of Arbidol in the clinical treatment of COVID-19 in humans was limited. Several small sample retrospective studies were reported [12, 13], but large, retrospective or prospective clinical studies were lacking.

Due to the lack of reliable markers, the monitoring of COVID-19 mainly relies on clinical observation. In infections caused by highly homologous viruses, such as SARS and MERS, lymphocytopenia and increased levels of inflammatory cytokines are typical laboratory abnormalities associated with disease severity [14, 15]. Decreased lymphocyte numbers and increased levels of inflammatory mediators, such as hypersensitive C reactive protein (hs-CRP) and interleukin-6 (IL-6) were also reported in COVID-19 patients [3, 4, 16]. Given the high mortality rate of severe COVID-19 cases, a better understanding of the clinical features may help identify reliable markers for monitoring the inflammatory response associated with COVID-19 progression.

In this study, we retrospectively analyzed 252 COVID-19 patients treated in our department and compared the outcome differences in patients treated with and without Arbidol. To evaluate the characteristics of clinically improved patients treated with Arbidol, the differences in combination treatments, and laboratory and immunological examinations between improved and non-improved patients treated with Arbidol were also analyzed.

## MATERIALS AND METHODS

### Study design and participants

This was a single center, retrospective study. The Cancer Center, Union Hospital, Tongji Medical College of Huazhong University of Science and Technology, which is only 600m straight-line distance from the Huanan seafood market was designated to receive patients with COVID-19 during the disease outbreak in Wuhan, China.

We retrospectively analyzed all patients diagnosed with COVID-19 in 4 inpatient wards from Feb. 13 to Feb. 29. 2020. The study inclusion criteria were: (1) age ≥ 18 years, (2) laboratory confirmed COVID-19 by viral nucleic acid test using real-time RT-PCR detection in samples taken from the respiratory tract of patients. All patients were followed up for 3 weeks after they were admitted to the hospital. This study was approved by the Ethics Committee of Union Hospital, Tongji Medical College, Huazhong University of Science and Technology (committee’s reference number 0049) and was performed in accordance with the World Medical Association Declaration of Helsinki and the Department of Health and Human Services Belmont Report.

### Data collection

Data were obtained from electronic medical record system, nursing records, and laboratory and radiology examination system of the Hospital. Demographic, clinical, laboratory, radiological, and treatment data were collected and analyzed by trained physicians. Missing or uncertain data in the records were clarified by direct communication with the patients or their family members.

Based on the China Guidelines for COVID-19 [11], COVID-19 was classified into four types: 1) mild type with slight clinical symptoms, no radiological imaging presentations of pneumonia; 2) common type with fever, respiratory symptoms and radiological imaging presentations of pneumonia; 3) severe type with any of the following: respiratory distress with RR> 30 times/min, oxygen saturation at rest <93%, or PaO2/FiO2<300 mmHg; 4) critical severe type with any of the following: respiratory failure needing mechanical ventilation, shock, or organ failure needing intensive care unit (ICU) intensive care.

All types of confirmed COVID-19 patients received antiviral treatments; most of them received Arbidol (200 mg three times daily). Other antiviral treatments included Oseltamivir (75 mg, two times daily), Ribavirin (500 mg, three times daily), and Interferon-α (5 million U, two times daily). Most patients received empirical or prophylactic antibiotic intervention. Most patients received traditional Chinese medicine for symptom relief and supportive care. Most patients underwent laboratory testing including routine blood, biochemical and coagulation tests, and immunological examinations every 3–7 days, and COVID-19 RT-PCR test every 3–5 days. Chest CT scans were performed for all patients at the time of admission and every 5–7 days after treatment.

### Outcomes

**Figure 1:**
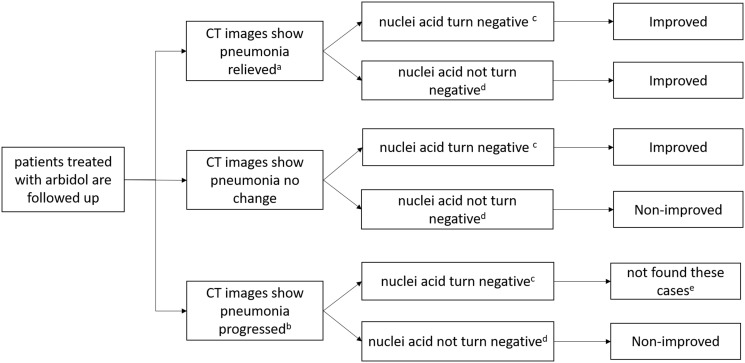
**Definition of clinically improved and non-improved.** a: including lesion areas absorbed, improved with reduced extent, decreased density and/or formation of fibrotic stripes. b: including lesion areas enlarged, and/or with increased density. c: means at least the last twice results of viral nucleic acid tests must be negative. d: means at least one of the last two results of viral nucleic acid test is still positive. e: we did not found patients like this, so remove this type.

The primary endpoint was the treatment outcome: the percentage of clinically improved or non-improved patients. The definition of improved and non-improved is shown in Figure 1. In brief, clinically improved cases were defined as patients with CT images demonstrating pneumonia relieve, or with CT images showing no change but having a negative COVID-19 RT-PCR test. Non-improved cases were defined as having CT images showing that pneumonia progressed, or having CT images with no change, and a positive RT-PCR tests. The differences in combination treatment, laboratory and immunological abnormalities between improved and non-improved patients treated with Arbidol were also evaluated. The evaluation criteria referred to common terminology criteria for adverse events (CTCAE v 4.0).

### Statistics

The statistical analysis was performed with GraphPad Prism 5.0 software. Data were presented as mean ± standard deviation (SD) or median and interquartile range (IQR). Continuous variables that did not follow normal distribution were compared by the Mann-Whitney *U* test. Proportions for categorical variables were compared by the chi-square test. Data from repeated measures were compared using the two-way ANOVA analysis. The significance *P* value was set at < 0.05 on both sides.

## RESULTS

**Figure 2:**
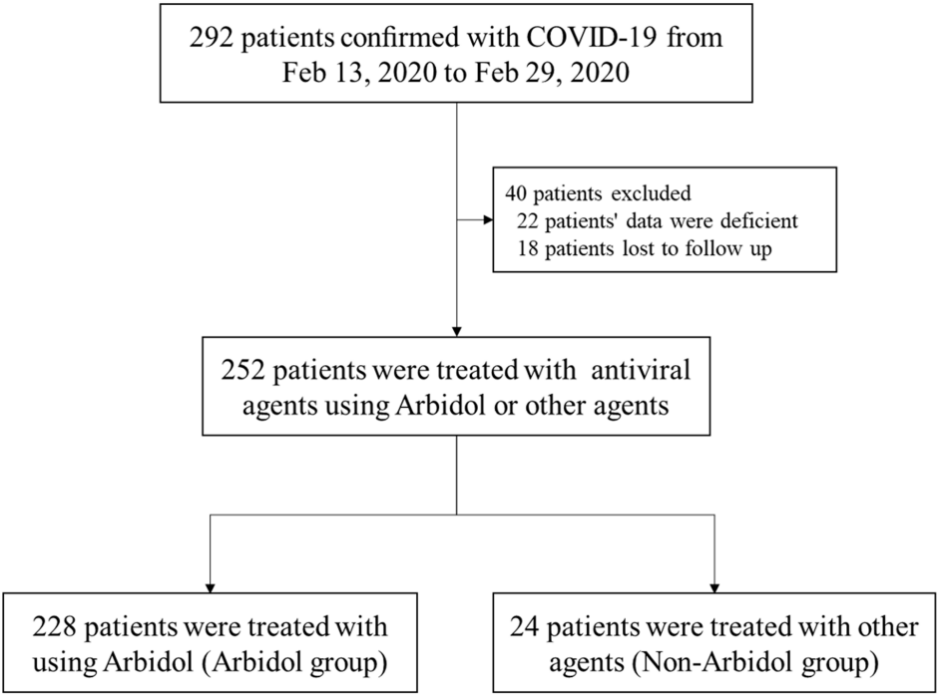
**Flowchart of patient cohort.** Inclusion or exclusion of patients according to their antiviral medications and subsequent follow-up records.

Between Feb 13, 2020 and Feb 29, 2020, 292 patients with COVID-19 were admitted to 4 inpatient wards of Cancer Center, Union Hospital, Tongji Medical College of Huazhong University of Science and Technology. 22 patients were excluded because of lack of data (e.g., a failure to complete laboratory or immunological tests), and another 18 patients were excluded because of the lack of a follow up. Finally, 228 cases in the Arbidol group and 24 cases in the No-Arbidol group were enrolled in this study (Figure 2).

Among the 252 enrolled patients, 106 (42.1%) were males and 146 (57.9%) were females. The median age was 65 years (range: 20-97 years, IQR: 56-69 years). There were 138 cases (54.8%) complicated with underlying diseases, including 107 cases (42.5%) with chronic cardiovascular diseases, 14 cases (5.6%) with chronic pulmonary diseases, 44 cases (17.5%) with diabetes mellitus, and 24 (9.5%) cases having other diseases. Fever (81%), cough (63.5%), fatigue (53.6%) and dyspnea (51.6%) were the most common symptoms (> 50%) in these patients. The 252 enrolled cases were classified as mild type (0 patients), common type (122 patients; 48.4%), severe type (122 patients; 48.4%), and critical severe type (8 patients; 3.2%). There were no mild type patients because only the common, severe, and critical severe types were hospitalized in our department. 236 (93.7%) cases received antibiotics treatment, 197 (78.2%) cases received traditional Chinese medicine, 30 (11.9%) cases received glucocorticoid treatment, and 17 (6.7%) cases received immunoglobulin therapy. There were no significant differences in age, sex, chronic medical illness, symptoms, disease type, and combination treatment between Arbidol and No-Arbidol groups (Table 1).

**Table 1:** Clinical characteristics of 252 patients confirmed with COVID-19.

**Characteristics**	**Total Patients** **(*n* = 252)**	**Arbidol group** **(*n* = 228)**	**No-Arbidol group** **(*n* = 24)**	***P* value**
Median (IQR) age (years)	65 (56–69)	65 (55.5–69)	65.5 (59.5–72)	0.2992
Sex				0.6941
male	106 (42.1%)	95 (41.7%)	11 (45.8%)	
female	146 (57.9%)	133 (58.3%)	13 (54.1%)	
Chronic medical illness				
chronic cardiovascular disease	107 (42.5%)	93 (40.8%)	14 (58.3%)	0.0981
chronic pulmonary disease	14 (5.6%)	12 (5.3%)	2 (8.3%)	0.5323
diabetes	44 (17.5%)	39 (17.1%)	5 (20.8%)	0.6472
others	24 (9.5%)	23 (10.1%)	1 (4.1%)	0.3472
Symptoms				
fever	204 (81%)	186 (81.6%)	18 (75%)	0.4350
cough	160 (63.5%)	142 (62.3%)	18 (75%)	0.2183
fatigue	135 (53.6%)	120 (52.6%)	15 (62.5%)	0.3565
dyspnea	130 (51.6%)	115 (50.4%)	15 (62.5%)	0.2607
myalgia	92 (36.5%)	86 (37.7%)	6 (25%)	0.2183
pharyngalgia	49 (19.4%)	42 (18.4%)	7 (29.2%)	0.2058
diarrhea	40 (15.9%)	37 (16.2%)	3 (12.5%)	0.6345
abdominal pain	33 (13.1%)	31 (13.6%)	2 (8.3%)	0.4672
headache	27 (10.7%)	26 (11.4%)	1 (4.1%)	0.2756
vomiting	18 (7.1%)	15 (6.6%)	3 (12.5%)	0.2840
Disease type				
mild	0	0	0	
common	122 (48.4%)	113 (49.6%)	9 (37.5%)	0.2607
severe	122 (48.4%)	109 (47.8%)	13 (54.2%)	0.5532
critically severe	8 (3.2%)	6 (2.6%)	2 (8.3%)	0.1297
Combination treatment				
Antibacterial agents	236 (93.7%)	215 (94.3%)	21 (87.5%)	0.1939
Traditional Chinese medicine	197 (78.2%)	181 (79.4%)	16 (66.7%)	0.1513
Glucocorticoid therapy	30 (11.9%)	28 (12.3%)	2 (8.3%)	0.5700
Immunoglobulin therapy	17 (6.7%)	15 (6.6%)	2 (8.3%)	0.7445

According to the definition of clinical improvement, 211 (83.7%) cases were judged to be clinically improved, and 41 (16.7%) cases were judged to be non-improved; 14 (5.6%) patients in the non-improved group progressed to death. There was a significant difference in clinical improvement rates between Arbidol and No-Arbidol groups (86.8% vs. 54.2%, *p <* 0.0001). In the common-type and severe-type groups, the Arbidol group had a higher improvement rate compared with No-Arbidol group (95.6% vs.66.6%, *p* = 0.0007, and 81.7% vs. 53.8%, *p* = 0.0207, respectively), but there was no significant difference in clinical improvement between Arbidol and No-Arbidol groups in the critically severe type of population (*p* = 0.5371) (Table 2).

**Table 2:** Outcomes of 252 patients confirmed with COVID-19.

**Outcomes**	**Total Patients** **(*n* = 252)**	**Arbidol group** **(*n* = 228)**	**No-Arbidol group** **(*n* = 24)**	***P* value**
Total				<0.0001
Improved	211 (83.7%)	198 (86.8%)	13 (54.2%)	
Non-improved	41 (16.3%)	30 (13.2%)	11 (45.8%)	
Common type				0.0007
Improved	114 (93.4%)	108 (95.6%)	6 (66.6%)	
Non-improved	8 (6.6%)	5 (4.4%)	3 (33.3%)	
Server type				0.0207
Improved	96 (78.7%)	89 (81.7%)	7 (53.8%)	
Non-improved	26 (21.3%)	20 (18.3%)	6 (46.2%)	
Critically severe type				0.5371
Improved	1 (12.5%)	1 (16.7%)	0	
Non-improved	7 (87.5%)	5 (83.3%)	2 (100%)	

In the Arbidol group, the median duration time of Arbidol was 10.5 days (range 3–21 days, IQR: 9–14 days). 215 (94.3%) cases received antibiotics treatment, 181 (79.4%) cases received traditional Chinese medicine, 28 (12.3%) cases received glucocorticoid treatment, and 15 (6.6%) cases received immunoglobulin treatment. As shown in Table 3, there were significant differences in Arbidol duration time and use of traditional Chinese medicine between the improved and non-improved cases (*p* = 0.0197, and *p* < 0.0001, respectively). The percentages of glucocorticoid and immunoglobulin therapy in the non-improved group were significantly higher than the improved group (both *p* < 0.0001), suggesting that glucocorticoid and immunoglobulin therapy may not improve prognosis.

**Table 3:** Combination treatments 228 patients treated with Arbidol.

**Treatments**	**Total Patients** **(*n* = 228)**	**Improved** **(*n* = 198)**	**Non-improved** **(*n* = 30)**	***P* value**
Median (IQR) duration of Arbidol treatment(days)	10.5 (9–14)	11 (10–14)	8 (7–14)	0.0115
Arbidol duration groups (days)				
≤5	14 (6.1%)	9 (4.5%)	5 (16.7%)	
6–10	100 (43.9%)	85 (42.9%)	15 (50.0%)	
11–15	88 (38.6%)	82 (41.4%)	6 (20%)	
16–20	24 (10.5%)	20 (10.1%)	4 (13.3%)	
>20	2 (0.9%)	2 (1.0%)	0	
Antibacterial agents	215 (94.3%)	186 (93.9%)	29 (96.7%)	0.5483
Traditional Chinese medicine	181 (79.4%)	162 (81.8%)	19 (63.3%)	0.0197
Glucocorticoid therapy	28 (12.3%)	11 (5.6%)	17 (56.7%)	<0.0001
Immunoglobulin therapy	15 (6.6%)	6 (3.0%)	9 (30.0%)	<0.0001

The laboratory test results of the Arbidol group are shown in Table 4. 86 (37.7%) patients presented with lymphocytopenia, 19 (8.3%) patients with neutropenia, 63 (27.6%) patients with anemia (low haemoglobin), and 17 (7.5%) patients with thrombocytopenia. There were 80 (35.1%) cases with increased levels of hs-CRP, 79 (34.6%) cases with increased D-dimer, and 74 (32.5%) cases with increased levels of lactate dehydrogenase (LDH). Liver dysfunction was found in 78 patients (34.2%), but most of them (59 patients) were level I degree or mild damage. Myocardial damage or renal dysfunction were rare (0.4%, and 2.2%, respectively). The non-improved group showed higher proportion of lymphocytopenia (76.7% vs.31.8%, *p* < 0.0001), anemia (53.3 vs. 23.7%, *p* = 0.0007), and thrombocytopenia (33.3 vs.3.5%, *p* < 0.0001) compared with the improved group. The non-improved group had also increased percentage of cases with increased hsCRP (66.7 vs. 30.3%, *p* < 0.0001), D-dimer (56.7 vs. 31.3%, *p* = 0.0065), and LDH (66.7 vs. 27.3%, *p* < 0.0001). The non-improved group exhibited much higher levels of hsCRP, D-dimer, and LDH, which indicated poor prognosis.

**Table 4:** Laboratory findings of 228 patients treated with Arbidol.

**Characteristics**	**Total Patients** **(*n* = 228)**	**Improved** **(*n* = 198)**	**Non-improved** **(*n* = 30)**	***P* value**
Lymphocytopenia (normal range 1.1–3.2 × 10^9^/L)	86 (37.7%)	63 (31.8%)	23 (76.7%)	<0.0001
0.5–1.0	73 (32.0%)	60 (30.3%)	13 (43.3%)	
<0.5	13 (5.7%)	3 (1.5%)	10 (33.3%)	
Neutropenia (normal range 1.8–6.3 × 10^9^/L)	19 (8.3%)	14 (7.1%)	5 (16.7%)	0.0764
1.50–1.79	7 (3.1%)	6 (3.0%)	1 (3.3%)	
1.00–1.49	9 (3.9%)	7 (3.5%)	2 (6.7%)	
0.50–0.99	3 (1.3%)	1 (0.5%)	2 (6.7%)	
Low hemoglobin (normal range 120–175 g/L)	63 (27.6%)	47 (23.7%)	16 (53.3%)	0.0007
90–119	52 (22.8%)	41 (20.7%)	11 (36.7%)	
60–89	11 (4.8%)	6 (3.0%)	5 (16.7%)	
Thrombocytopenia (normal range 100–350 × 10^9^/L)	17 (7.5%)	7 (3.5%)	10 (33.3%)	<0.0001
75–99	7 (3.1%)	6 (3.0%)	1 (3.3%)	
50–74	2 (0.9%)	0 (0.0%)	2 (6.7%)	
25–49	6 (2.6%)	1 (0.5%)	5 (16.7%)	
<25	2 (0.9%)	0 (0.0%)	2 (6.7%)	
Increased hypersensitive C reactive protein (normal range <4.0mg/L)	80 (35.1%)	60 (30.3%)	20 (66.7%)	0.0001
4–9.9	20 (8.8%)	19 (9.6%)	1 (3.3%)	
10–19.9	18 (7.9%)	15 (7.6%)	3 (10.0%)	
20–99.9	32 (14%)	23 (11.6%)	9 (30.0%)	
100–200	10 (4.4%)	3 (1.5%)	7 (23.3%)	
Increased D-dimer (normal range <0.5mg/L)	79 (34.6%)	62 (31.3%)	17 (56.7%)	0.0065
0.5–0.9	20 (8.8%)	20 (10.1%)	0	
1.0–1.9	26 (11.4%)	20 (10.1%)	6 (20.0%)	
2.0–4.9	29 (12.7%)	22 (11.1%)	7 (23.3%)	
5.0–10.0	4 (1.8%)	0	4 (13.3%)	
Increased lactate dehydrogenase (normal range 109–245U/L)	74 (32.5%)	54 (27.3%)	20 (66.7%)	<0.0001
246–299	41 (18%)	34 (17.2%)	7 (23.3%)	
300–399	17 (7.4%)	13 (6.6%)	4 (13.3%)	
400–1000	16 (7%)	7 (3.5%)	9 (30.0%)	
Increased alanine aminotransferase (normal range 5–40U/L)	78 (34.2%)	65 (32.8%)	13 (43.3%)	0.2584
41–99	59 (25.9%)	50 (25.3%)	9 (30.0%)	
100–199	17 (7.5%)	14 (7.1%)	3 (10.0%)	
200–500	2 (0.9%)	1 (0.5%)	1 (3.3%)	
Increased Creatinine (normal range 44–133umol/L)	1 (0.4%)	0	1 (3.3%)	0.0100
134–200	1 (0.4%)	0	1 (3.3%)	
Increased hypersensitive troponin I (normal range <26.2ng/L)	5 (2.2%)	0	5 (16.7%)	<0.0001
26.3–50	5 (2.2%)	0	5 (16.7%)	

The data of immunological examinations in the Arbidol group are shown in Table 5. 55 (24.1%) patients had increased levels of IL-2, 84 (36.8%) patients had increased levels of IL-4, and 63 (27.6%) patients had slightly increased levels of IL-10. Most of the patients (181 cases, 79.4%) showed increased levels of IL-6, and 80 (35.1%) cases showed substantially increased levels of IL-6 (>100 pg/L). The proportions of CD3 + T cells, CD4 + T cells, and the ratio of CD4/CD8 + T cells were slightly increased in some patients (11.0%, 27.6%, and 20.6%, respectively). Increased levels of CD8 + T cells were rare (1.3%). The non-improved group showed significantly more proportions of high levels of IL-6 (96.7 vs.76.8%, *p* = 0.0120) and IL-10 (56.7 vs. 23.2%, *p* < 0.0001) compared with the improved group. Conspicuously, IL-6 was increased in 96.7% of patients in the non-improved group, suggesting that it may be an important prognostic indicator.

**Table 5:** Immunological examinations of 228 patients treated with Arbidol.

**Characteristics**	**Total Patients** **(*n* = 228)**	**Improved** **(*n* = 198)**	**Non-improved** **(*n* = 30)**	***P* value**
Increased interleukin-2 (normal range 0.1–4.1pg/ml)	55 (24.1%)	44 (22.2%)	11 (36.7%)	0.0848
4.2–5.9	44 (19.3%)	35 (17.7%)	9 (30.0%)	
6.1–10	11 (4.8%)	9 (4.5%)	2 (6.7%)	
Increased interleukin-4 (normal range 0.1–3.2pg/ml)	84 (36.8%)	71 (35.9%)	13 (43.3%)	0.4290
3.3–4.9	62 (27.2%)	52 (26.3%)	10 (33.3%)	
5.0–7.0	22 (9.6%)	19 (9.6%)	3 (10.0%)	
Increased interleukin-6 (normal range 0.1–2.9pg/ml)	181 (79.4%)	152 (76.8%)	29 (96.7%)	0.0120
3.0–9.9	25 (11%)	24 (12.1%)	1 (3.3%)	
10.0–99.9	76 (33.3%)	60 (30.3%)	16 (53.3%)	
100.0–499.9	59 (25.9%)	49 (24.7%)	10 (33.3%)	
500–1000.0	21 (9.2%)	19 (9.6%)	2 (6.7%)	
Increased interleukin-10 (normal range 0.1–5.0pg/ml)	63 (27.6%)	46 (23.2%)	17 (56.7%)	<0.0001
5.1–9.9	59 (25.9%)	44 (22.2%)	15 (50.0%)	
10.0–20.0	4 (1.8%)	2 (1.0%)	2 (6.7%)	
Increased CD3 + T cells (normal range 58.2–84.2%)	25 (11.0%)	22 (11.1%)	3 (10.0%)	0.8560
84.3–89.9	19 (8.3%)	17 (8.6%)	2 (6.7%)	
90.0–95.0	6 (2.6%)	5 (2.5%)	1 (3.3%)	
Increased CD4 + T cells (normal range 25.3–51.4%)	63 (27.6%)	55 (27.8%)	8 (26.7%)	0.8991
51.5–59.9	47 (20.6%)	42 (21.2%)	5 (16.7%)	
60.0–70.0	16 (7%)	13 (6.6%)	3 (10.0%)	
Increased CD8 +T cells (normal range 14.2–38.9%)	3 (1.3%)	2 (1.0%)	1 (3.3%)	0.2980
39–50	3 (1.3%)	2 (1.0%)	1 (3.3%)	
Increased CD4/CD8 ratio (normal range 0.41–2.72)	47 (20.6%)	37 (18.7%)	10 (33.3%)	0.0646
2.73–2.99	10 (4.4%)	9 (4.5%)	1 (3.3%)	
3.00–3.99	22 (9.6%)	17 (8.6%)	5 (16.7%)	
4.00–5.0	15 (6.6%)	11 (5.6%)	4 (13.3%)	

**Figure 3:**
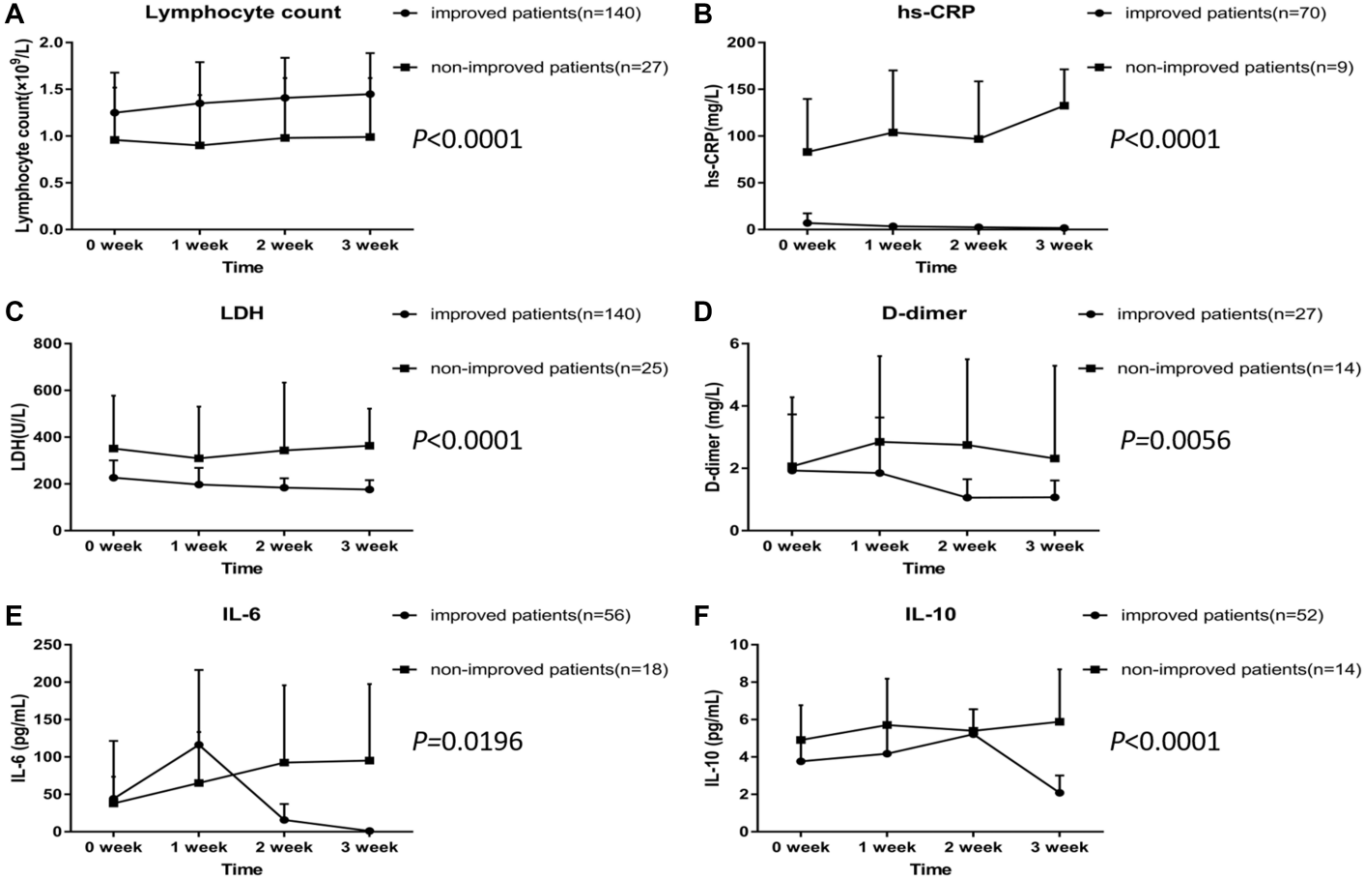
**Dynamic profile of laboratory and immunological parameters in COVID-19 patients treated with Arbidol.** (**A**) Lymphocyte count. (**B**) hs-CRP. (**C**) LDH. (**D**) D-dimer. (**E**) IL-6. (**F**) IL-10.

The changes of laboratory and immunological parameters, including lymphocyte counts, hsCRP, LDH, D-dimer, IL-6, and IL-10 levels were monitored (at 1-week intervals, in some patients) from day 1 to day 21 after hospitalization. As shown in Figure 3, non-improved patients showed lower lymphocyte counts, and higher levels of hsCRP, LDH, D-dimer, IL-6, and IL-10 compared with improved patients. The means of hsCRP, LDH, D-dimer, and IL-10 declined gradually during the treatment in the improved patients. The means of IL-6 increased in the first week, and then decreased in the improved patients. In contrast, the levels of lymphocyte count, hsCRP, LDH, D-dimer, IL-6, and IL-10 did not change in the non-improved patients over time.

## DISCUSSION

Our study showed that the overall clinical improvement rate after Arbidol treatment was 86.8%, which was significantly higher than in COVID-19 patients not treated with Arbidol (54.2%). No serious adverse effects were observed in the Arbidol group. For common and severe disease types, the clinical improvement rates in Arbidol group were 95.6% and 81.7%, significantly higher than in Non-Arbidol group (66.6% and 53.8%). There was no significant difference in clinical improvement in the critically severe type, as the number of critically severe cases was small, and it was more important for critically severe cases to manage underlying complications. Some observational studies indicated that Arbidol might be effective in COVID-19 treatment [12, 13]. A randomized controlled trial, which enrolled 100 patients demonstrated that Arbidol, compared to KALETRA, was associated with clinical and laboratory improvements, including peripheral oxygen saturation, need for ICU admission, duration of hospitalization, chest CT, white blood cell counts, and erythrocyte sedimentation rate [17]. However, large sample size randomized controlled trials of Arbidol in COVID-19 were lacking. Our study suggested that Arbidol might be effective in the treatment of COVID-19 patients with tolerable adverse effects, especially in common and severe disease types.

The recommended duration time for Arbidol to treat influenza was 5 days [7, 8]. However, we found that improved patients had a longer duration time of Arbidol treatment compared with non-improved patients. Another randomized controlled trial also suggested longer Arbidol treatment (200 mg three times daily, 7 to 14 days) based on the severity of disease [17]. Since the time of Arbidol treatment likely contributes to clinical improvement of COVID-19 patients, we suggest that the time of Arbidol treatment of COVID-19 is extended, as long as the side effects can be tolerated.

Most improved patients in our study also received traditional Chinese medicine. Application of traditional Chinese medicine in the treatment of COVID-19 has been inspired by the treatment of SARS-CoV in 2002 in Guangdong Province in China [18]. A recent study of four COVID-19 patients reported that three patients were significantly improved by using traditional Chinese medicine combined with western medicine [19]. The purpose of traditional Chinese medicine treatment is to relieve symptoms and enhance physical fitness. Since the treatment is based primarily on an overall analysis of the individual patient's symptoms, each patient receives different combination and regimen of Chinese herbs. Although there were statistical differences between improved and non-improved patients regarding the use of traditional Chinese medicine in this study, the number of patients who did not receive traditional Chinese medicine was small. The precise effect of traditional Chinese medicine in COVID-19 treatment needs to be analyzed in future by a strictly designed prospective randomized controlled study.

The use antibiotics in COVID-19 treatment was controversial. Considering the high probability of secondary bacterial infections in COVID-19 patients, most physicians recommend the empirical or prophylactic use of antibiotics [1–4]. In our study, no significant difference was found between the improved and non-improved patients regarding the use of antibiotic. Since the number of COVID-19 patients who did not receive antibiotics in our study was small, the results should be confirmed by large sample clinical studies.

Our study showed that non-improved patients had higher rates of lymphocytopenia, anemia, and thrombocytopenia, and increased levels of hsCRP, LDH, and D-dimer than improved patients. Lymphocytopenia was also considered as the main feature of SARS and MERS cases [20–23]. It is possible that the virus induces lymphocyte apoptosis and destruction [24, 25]. Apart from lymphocytopenia, increased rates of anemia and thrombocytopenia were also observed in non-improved patients in this study. We speculate that a disordered immune response during COVID-19 progression may directly impair red blood cells and platelets, resulting in their decline. hsCRP is the predominant acute phase protein in infection-related inflammation, and high serum LDH may reflect cell damage and inflammation in lung tissues [26, 27]. High levels of D-dimer reflect coagulation disorder, which may be related to persistent inflammatory response. The levels of these markers were similar to previous studies [2–4], suggesting that they might be important prognostic indicators in COVID-19. Our data showed that the levels of hsCRP, LDH, and D-dimer decreased after treatment in improved patients but did not change or increased in non-improved patients, indicating that changes in these markers might be associated with severity and disease course of COVID-19.

Previous studies have suggested that inflammatory cytokine storm was associated with the severity of COVID-19 infection [28]. Our data showed that non-improved patients had significantly higher levels of IL-6 and IL-10 compared with improved patients. IL-6 is produced by a variety of cells in the lung parenchyma, including alveolar macrophages, type II pneumocytes, T lymphocytes, and lung fibroblasts. IL-6 is an acute phase inflammatory cytokine, and its circulating levels reflect the lung inflammation [29, 30]. Our finding that non-improved patients had significantly increased IL-6 levels is consistent with other studies demonstrating that high IL-6 levels correlate with severity of COVID-19 [31], and that increased plasma and bronchoalveolar IL-6 levels are associated with lung injury and prolonged mechanical ventilation, organ dysfunction, morbidity and mortality in lung diseases [32, 33]. IL-10 is produced by a variety of cells, including B cells, monocytes, DCs, NK cells, and T cells. In influenza infection, IL-10 is highly abundant, especially during the adaptive immune response [34]. Our data showed that improved patients had elevated levels of IL-6 and IL-10 in early stages, but these levels declined in later stages. These results indicated that IL-6 and IL-10 were increased at the beginning of COVID-19 infection, but their levels decreased during treatment and correlated with disease outcome; thus, these two cytokines might serve as early diagnostic and prognostic markers of COVID-19. In addition, targeting IL-6 may ameliorate the cytokine storm-related symptoms in severe COVID-19 cases. Indeed, promising therapeutic effects of targeting IL-6 were recently reported in severe COVID-19 patients [35].

In summary, our data demonstrated that Arbidol was effective in the treatment of COVID-19 patients and had tolerable adverse effects. Thus, Arbidol may represent a cost-effective pharmacological approach affordable for developing countries in urgent need for effective antiviral therapies. In addition, our data suggested that lymphocytopenia, and increased levels of hsCRP, D-dimer, LDH, IL-6, and IL-10 were associated with severity and disease course of COVID-19 and might indicate a poor therapeutic efficacy.

### Ethics approval and consent to participate

This study was approved by the Ethics Committee of Union Hospital, Tongji Medical College, Huazhong University of Science and Technology and conformed to the principles set out in the WMA Declaration of Helsinki and the Department of Health and Human Services Belmont Report. The committee’s reference number was 0049.
